# A Novel Dynamic Neonatal Blood-Brain Barrier on a Chip

**DOI:** 10.1371/journal.pone.0142725

**Published:** 2015-11-10

**Authors:** Sudhir P. Deosarkar, Balabhaskar Prabhakarpandian, Bin Wang, Joel B. Sheffield, Barbara Krynska, Mohammad F. Kiani

**Affiliations:** 1 Department of Mechanical Engineering, Temple University, Philadelphia, Pennsylvania, 19122, United States of America; 2 Biomedical Technology, CFD Research Corporation, Huntsville, Alabama, 35806, United States of America; 3 Department of Biomedical Engineering, Widener University, Chester, Pennsylvania, 19013, United States of America; 4 Department of Biology, Temple University, Philadelphia, Pennsylvania, 19122, United States of America; 5 Shriners Hospitals Pediatric Research Center, Temple University School of Medicine, Philadelphia, Pennsylvania, 19122, United States of America; Hungarian Academy of Sciences, HUNGARY

## Abstract

Studies of neonatal neural pathologies and development of appropriate therapeutics are hampered by a lack of relevant *in vitro* models of neonatal blood-brain barrier (BBB). To establish such a model, we have developed a novel blood-brain barrier on a chip (B^3^C) that comprises a tissue compartment and vascular channels placed side-by-side mimicking the three-dimensional morphology, size and flow characteristics of microvessels *in vivo*. Rat brain endothelial cells (RBEC) isolated from neonatal rats were seeded in the vascular channels of B^3^C and maintained under shear flow conditions, while neonatal rat astrocytes were cultured under static conditions in the tissue compartment of the B^3^C. RBEC formed continuous endothelial lining with a central lumen along the length of the vascular channels of B^3^C and exhibited tight junction formation, as measured by the expression of zonula occludens-1 (ZO-1). ZO-1 expression significantly increased with shear flow in the vascular channels and with the presence of astrocyte conditioned medium (ACM) or astrocytes cultured in the tissue compartment. Consistent with *in vivo* BBB, B^3^C allowed endfeet-like astrocyte-endothelial cell interactions through a porous interface that separates the tissue compartment containing cultured astrocytes from the cultured RBEC in the vascular channels. The permeability of fluorescent 40 kDa dextran from vascular channel to the tissue compartment significantly decreased when RBEC were cultured in the presence of astrocytes or ACM (from 41.0±0.9 x 10^−6^ cm/s to 2.9±1.0 x 10^−6^ cm/s or 1.1±0.4 x 10^−6^ cm/s, respectively). Measurement of electrical resistance in B^3^C further supports that the addition of ACM significantly improves the barrier function in neonatal RBEC. Moreover, B^3^C exhibits significantly improved barrier characteristics compared to the transwell model and B^3^C permeability was not significantly different from the *in vivo* BBB permeability in neonatal rats. In summary, we developed a first dynamic *in vitro* neonatal BBB on a chip (B^3^C) that closely mimics the *in vivo* microenvironment, offers the flexibility of real time analysis, and is suitable for studies of BBB function as well as screening of novel therapeutics.

## Introduction

Blood-brain barrier (BBB) is a physical and functional barrier formed by the brain vascular endothelial cells and perivascular cells [1, 2]. It is highly effective and selective to the passage of molecules from the blood to the brain tissue, and is important for the maintenance of normal function of the brain in adults and neonates [3–7]. Although the significance of the BBB in the initiation and progression of neonatal neural pathologies, e.g. neurodevelopmental injuries, has been recognized, research in this area has been hampered by a lack of relevant *in vitro* models of neonatal BBB [8]. Small animal models often used to study the permeability across BBB have the advantage of studying the brain in its natural environment [9–11]. However, such *in vivo* studies are expensive, lengthy and difficult to perform especially in neonatal animals. Therefore, there has been a long-standing interest in the development of *in vitro* BBB models that could mimic the neonatal *in vivo* BBB microenvironment. Traditional *in vitro* BBB models use static cell-based assays in a transwell type apparatus to measure permeability of various tracers which provide suitable models for both drug permeability studies and physiological and pathological experiments [12, 13]. Transwell based BBB models have also been improved to approximate several important aspects of the *in vivo* BBB including high electrical resistance and realistic cytoarchitecture [12–16]. Nevertheless, these *in vitro* BBB models often lack realistic morphological (e.g. realistic microvascular size and tube-like structure of vascular channels) and functional (e.g. physiological shear flow in the vascular compartment) features that allow for the development of a realistic *in vivo*-like microenvironment. Furthermore, two-dimensional monocultures of primary adult endothelial cells used in simple BBB *in vitro* models over time lose many of the characteristics of the *in vivo* phenotype, e.g. tight junction formation. These observations suggest that a proper microenvironment such as factors secreted by the perivascular cells and/or realistic shear forces from blood flow is required to maintain an optimally functioning neonatal *in vitro* BBB.

More recently, newer *in vitro* BBB models have been developed that attempt to incorporate some of the important features of the *in vivo* brain microenvironment. These two dimensional models are developed using either a monoculture of adult endothelial cells, co-culture of endothelial cells with glial cells, or the factors secreted by the glial cells in either a static or a dynamic flow based configuration [17–23]. Of these, the DIV-BBB and NDIV-BBB models developed by Cucullo et al. represent a new class of dynamic *in vitro* BBB models that incorporate shear flow in addition to the presence of glial cells. However, these devices use large fiber diameter (>600 μm) which necessitates unrealistically high flow rates to maintain physiological shear and alters the balance of convective and diffusive transport. The larger size of the device also leads to larger requirement of consumables. To minimize the large volumes of samples, researchers have adapted to microfluidics based approaches for development of the BBB model [24, 25]. However, these microfluidic models still employ transwell membranes that do not allow real-time visualization of the BBB function and lack realistic microvascular geometries. Furthermore, neonatal and adult BBB have been found to exhibit significant differences in terms of their structure and function, thus neonatal endothelial and perivascular cells are required to accurately represent the neonatal BBB [26–30]. In a recent study a transwell based static *in vitro* model of a neonatal BBB was developed for the first time [31]. Although this study used brain capillary endothelial cells isolated from neonatal rat brain, this model still lacks the shear flow and the realistic three-dimensional microvascular geometry that are essential for a dynamic, physiologically realistic *in vitro* BBB model.

To overcome the above mentioned limitations, in a previous study, our group developed a framework for a microfluidic model of the BBB using a well characterized adult rat endothelial cell line (RBE4) [32]. In the present study, we report on the development of a novel dynamic neonatal blood-brain barrier on a chip (B^3^C) constructed with a tissue compartment and microvascular channels that mimic the three-dimensional morphology, size, and flow characteristics of microvessels *in vivo*. For the first time, B^3^C allows for the co-culture of primary neonatal brain endothelial cells and astrocytes in communication through porous interface, leading to a realistically tight barrier, which mimics the neonatal *in vivo* BBB. The side-by-side placement of optically clear vascular channels and the tissue compartment in B^3^C allows for real-time visualization and direct measurement of the dynamic processes taking place within the neonatal BBB which is a significant advantage when compared with transwell or other membrane based *in vitro* models of the BBB.

## Materials and Methods

### Materials

Endothelial basal medium-2 (EBM-2) and Astrocyte Growth Medium (AGM) bullet kit were from Lonza Inc., Walkersville MD. Rat Plasma Fibronectin was from EMD Millipore, Billerica MA. TrypLE Select, Rat Tail Collagen Type I, Recovery Cell Culture Freezing Medium, Alexa Fluor^®^ 488 Phalloidin, 10% Normal Goat Serum, Texas Red-dextran (40 kDa), Hoechst 33342 nuclear stain were from Life Technologies Corporation, Carlsbad CA. Hyclone Phosphate Buffered Saline (PBS), tissue culture treated T75 culture flasks, BD Tuberculin Slip Tip 1ml syringe were from Fisher Scientific, Pittsburgh PA. 99.9% Methanol, Glacial Acetic Acid, 96% Paraformaldehyde and Draq5 Fluorescent Probe were from Thermofisher Scientific, Rockford IL. Premium Select FBS was from Atlanta Biologics, Lawrenceville GA. Bovine Plasma Derived Serum (BPDS) was from Animal Technologies Inc., Tyler TX. Tygon tubing (Cat. Number: AAD04103) was from Saint Gobbin PPL Corp., Valley Forge PA. 1000 μl gas tight syringes were from Hamilton Laboratory Products, Reno NV.

### Antibodies

Rabbit polyclonal anti-ZO-1 antibody (Cat. Number: 402300), Mouse monoclonal anti-CD11b antibody (Cat. Number: MA181606), Alexa Fluor^®^ 594 Goat anti-rabbit IgG secondary antibody (Cat. Number: A-11012), Alexa Fluor^®^ 488 Goat anti-rabbit IgG secondary antibody (Cat. Number: A-11008), Alexa Fluor^®^ 488 Goat anti-mouse IgG secondary antibody (Cat. Number: A11029) were from Life Technologies Corporation, Carlsbad CA. Rabbit polyclonal anti-GFAP antibody (Cat. Number: ab7260) was from Abcam Plc., Cambridge MA.

### Fabrication of B^3^C

**Fig 1:**
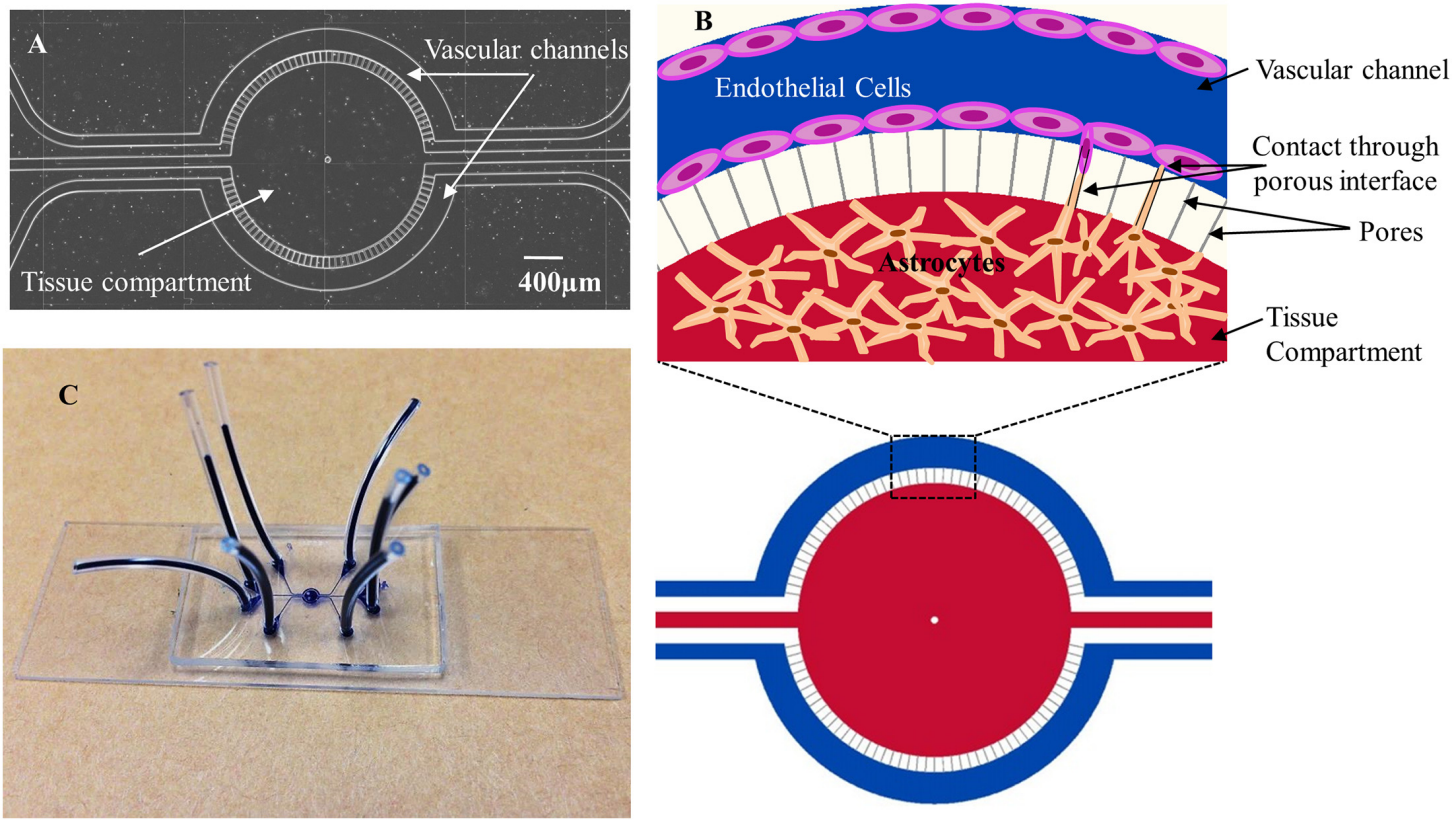
Schematic illustration and images of neonatal blood-brain barrier on a chip (B^3^C). Schematic illustration of B^3^C showing the tissue compartment in the center of the device surrounded by two independent vascular channels with flow access openings. The dimensions of vascular channels are 200 μm x 100 μm x 2762 μm (width x height x length) and the dimensions of tissue compartment are 1575 μm x 100 μm (diameter x height). Vascular channels are in communication with the tissue compartment through a series of 3μm porous interface (pore dimensions are: 3μm x 3μm x 100 μm, width x height x length, spaced every 50 μm) along the length of the vascular channels (A). Schematic illustration of cell culture in B^3^C device showing one of two vascular channels (blue) with endothelial cells lining the channel walls, the tissue compartment (red) containing astrocytes, and the porous interface (white) separating the vascular channel and tissue compartment (B). The B^3^C device is assembled on a microscope glass slide with plastic tubes (dark blue) allowing access to individual vascular channels and the tissue compartment (C).

To fabricate the microfluidic neonatal BBB on a chip (B^3^C), a photomask of the design shown in Fig 1A was created and soft photolithography was used to fabricate the final B^3^C model shown in Fig 1C on a microscope slide as described previously [32]. Briefly, Sylgard 184 Polydimethylsiloxane (PDMS) was prepared according to manufacturer’s (Dow Corning, Midland, MI) instructions and was poured over the developed master in a 150 mm Petri dish which was degassed for 15 min. The polymer was then allowed to cure overnight in an oven at 65°C. Inlet and outlet holes were punched using a 1.5 mm punch. The bonding surfaces of the PDMS and glass slide were plasma treated in a plasma generator (Harrick Scientific, Ithaca, NY). The assembly was heated at 75°C for 10 min to achieve a seal between the PDMS and glass yielding the complete device. The resulting B^3^C comprises of a disposable optically clear polydimethylsiloxane (PDMS) microfluidic chip containing a tissue and vascular channels [32, 33]. B^3^C is designed to allow culturing of brain cells in a tissue compartment and endothelial cells in two independent vascular channels with dimensions of 200 μm x 100 μm x 2762 μm (width x height x length) encompassing the tissue compartment. The tissue compartment and vascular channels are separated by an interface with a series of 3 μm pores along the length of the vascular channels, replacing the use of membranes in conventional models. Vascular channels and the tissue compartment in B^3^C are fabricated from optically clear PDMS and their side-by-side placement permits simultaneous real-time visualization of both compartments. The porous interface allows for biochemical and cellular communication between the two compartments. The size of the vascular channel is in the range of diameters observed in neonatal rats evaluated using a cranial window model and fluorescence microscopy [11].

### Cell Cultures

All experiments involving animals were carried out in strict accordance with the recommendations in the Guide for the Care and Use of Laboratory Animals of the National Institutes of Health. The protocol was approved by the Institutional Animal Care and Use Committee of Temple University (Permit Number: 4179). All efforts were made to minimize suffering. Sprague-Dawley (SD) rats were obtained from Taconic Farms (Hudson, NY). Primary cultures of neonatal rat brain capillary endothelial cells (RBEC) were obtained from two week old SD rats of either sex, with a modification of previously described protocols [31]. Rats were euthanized by CO_2_ followed by decapitation; brains were removed and placed in ice-cold phosphate-buffered saline (PBS). After removing the meninges, the cortices were minced into small pieces, gently dissociated by pipetting and digested in a mixture of collagenase/dispase (270 U/mL and 0.1%, respectively, GibcoBRL) and DNAse (10 U/mL, Sigma-Aldrich) in DMEM for 1.5 H at 37°C. After digestion, the microvessels were separated by centrifugation in 20% bovine serum albumin (BSA) in DMEM (1000 x g for 15 min). The microvessels obtained in the pellet were further digested in collagenase/dispase mixture for 1 H at 37°C. Capillary fragments were isolated by filtering through 10 μm nylon filter before plating on collagen type IV coated dishes (5 μg/cm^2^, Sigma-Aldrich). RBEC cultures were maintained in endothelial cell basal medium (Lonza Inc.) supplemented with 20% Bovine plasma-derived serum (BPDS), penicillin and streptomycin 100 U/mL & 100 μg/ml respectively (Mediatech Inc.) containing puromycin (4 μg/ml, Sigma-Aldrich) (allows selective growth of endothelial cells) and cultured for two days [34]. To remove the puromycin, cells were washed 3 times with fresh endothelial cell basal medium and cultured in endothelial cell basal medium (Lonza Inc.) supplemented with 20% Bovine plasma-derived serum, 100 μg/mL penicillin/streptomycin (Mediatech Inc.) containing basic Fibroblast Growth Factor (2 ng/mL; human recombinant; Invitrogen) and hydrocortisone (500 ng/mL; Sigma-Aldrich) until 80% -90% confluent. For subsequent RBEC culture, cells were cultured in flasks coated with rat collagen type I (5 μg/cm^2^). Cells from passage 3 were used in experiments.

Primary cultures of neonatal rat astrocytes were obtained from the cerebral cortex of two week old SD rats according to previously described methods [31]. In brief, after removing the meninges and blood vessels, the forebrains were minced and gently dissociated by repeated pipetting in DMEM containing 10% fetal bovine serum (FBS) (Atlanta Biologicals) and 100 μg/mL penicillin/streptomycin (Mediatech Inc.) and filtered through 70 μm cell strainer. After centrifugation, cells were re-suspended in DMEM containing 10% FBS and cultured in 75 cm^2^ tissue culture flasks at 37°C. Cell medium was changed every 2–3 days. The non-adherent cells were removed after 10–14 days in culture by vigorous shaking of the flask and the adherent astrocytes were thoroughly washed with PBS, trypsynized and seeded into new culture flasks. The purity of astrocytes (from passage 2) was confirmed using immunostaining for GFAP (an astrocytic marker) and CD11b (microglial cell marker) as shown in S4 Fig. For passaging, cells were cultured at the density of 5,000 cells per cm^2^ in astrocyte growth medium (AGM, Lonza Inc.), subconfluent growing cells were collected by washing the cells with PBS, centrifuged and then dissociated with trypsin followed by inactivation with medium containing serum. Cells from passage 2 were used in experiments.

For collection of astrocyte conditioned medium (ACM), neonatal rat astrocytes were cultured in astrocyte growth medium (AGM, Lonza Inc.) in T75 tissue culture flasks (Thermo Fisher Scientific Inc.) until reaching 95–100% confluence. Then, the medium was replaced with RBEC medium containing 2% BPDS. The cells were incubated in RBEC medium for 48 hours and then ACM was collected, filtered using 0.2 μm syringe filter (Millipore, SLMP025SS) and stored at -20°C until used in experiments. For all experiments using ACM, a 50:50 volume mixture of ACM and RBEC medium was used.

Adult rat brain endothelial cells (adult RBEC) isolated from 8–10 week old Sprague Dawley rats were purchased from Cell Applications, San Diego CA.

### RBEC and Astrocyte Culture in B^3^C

Prior to endothelial cell seeding, vascular channels of B^3^C were coated with rat fibronectin (40 μg/cm^2^, EMD Millipore) at 4°C overnight. RBEC at a concentration of 3–5x10^7^ cells/ml of RBEC medium were seeded at a flow rate of 10 μl/min in the fibronectin coated vascular channels using a syringe mounted on a programmable PHD Ultra syringe pump (Harvard Apparatus, Holliston MA). The B^3^C inlets were clamped when the RBEC reached a density of 80–90% in the vascular channels and the B^3^C was transferred to a CO_2_ incubator at 37°C to allow the cells to attach in the absence of flow. After 4–5 hours of incubation, the medium in the vascular channels was replaced with fresh RBEC medium or RBEC medium containing 50% ACM and flow was started at a rate of 0.01 μl/min (i.e. shear stress of 3.8x10^−3^ dynes/cm^2^) using syringes mounted on a PHD Ultra syringe pump placed adjacent to the incubator. B^3^C was connected to these syringes using Tygon tubing (Saint Gobain, USA; Cat. No. AAD04103), where ~75% of the total tube length of 12–18 inches resides inside the incubator thus warming up the media to 37°C before entering the B^3^C. Flow in the vascular channels was maintained for 5 days while syringes containing old medium were replaced with syringes containing fresh medium every 2–3 days. At the end of 5 days, cells in the vascular channels were either fixed with methanol or paraformaldehyde for immunofluorescence staining or the permeability was measured in cultured cells as described below.

For co-culture of RBEC with rat astrocytes, the vascular channels and the tissue compartment were coated with rat fibronectin (40 μg/cm^2^ and 20 μg/cm^2^, respectively, EMD Millipore). The device was kept at 4°C overnight before cell seeding was carried out. Prior to cell seeding the B^3^C was brought to room temperature and vascular channels were filled with AGM containing 20% FBS and the device was transferred to the CO_2_ incubator at 37°C. Subsequently, rat astrocytes from confluent cultures were harvested using TrypLE Select (Life Technologies Corp.) and resuspended in AGM containing 20% FBS at a concentration of 1.5–2.0x10^7^ cells/ml. This cell suspension was injected into the tissue compartment of B^3^C at a flow rate of 10 μl/min using a syringe mounted on a programmable PHD Ultra syringe pump (Harvard Apparatus, Holliston MA) as described above. When astrocytes in the tissue compartment reached a cell density of 50–60%, the access to the tissue compartment was closed and the device was returned to the CO_2_ incubator at 37°C for overnight incubation. On the next day, the vascular channels were coated again with rat fibronectin (40 μg/cm^2^, EMD Millipore) and maintained for about an hour at 37°C prior to seeding. Next, the medium in the entire device was replaced with RBEC medium containing 20% FBS and RBEC were seeded onto fibronection coated inner surface of vascular channels as described above. RBEC medium was flowed over RBEC cultured in vascular channels at a flow rate of 0.01 μl/min (i.e. shear stress of 3.8x10^-3^ dynes/cm^2^) and the astrocytes in the tissue compartment were maintained under static conditions in RBEC medium. The syringes were covered with aluminium foil to avoid exposure of the medium to the light.

### Measurement of Permeability and Electrical Resistance in B^3^C

For permeability measurements, B^3^C was mounted on a fluorescence microscope (Nikon TE200) equipped with an automated stage, and images were acquired using ORCA Flash 4 camera (Hamamatsu Corp., USA). Prior to the analysis of permeability, the vascular channel was connected to a Hamilton gas tight syringe filled with Texas Red 40 kDa dextran (25 μM in RBEC culture medium) mounted on a syringe pump (PhD Ultra Syringe pump, Harvard Apparatus). A syringe warmer was used to keep the culture medium in B^3^C at a physiological temperature. Nikon’s NIS Elements software (Nikon Instruments Inc., Melville, NY) was used to control the microscope stage and the camera settings. The permeability was measured in B^3^C by imaging Texas Red 40 kDa dextran injected into the vascular channel. Fluorescence images of the entire device were captured at an exposure of 90 ms every minute for 2 hours while dextran was flowing through the vascular channel at a flow rate of 0.2 μl/min (i.e. shear stress of 7.6x10^−2^ dynes/cm^2^) at a concentration of 25 μM in RBEC culture medium. B^3^C is made from an optically clear PDMS, which is assembled on a microscope slide to monitor the passage of fluorescent 40 kDa dextran from vascular channels to the tissue compartment over time. Permeation of 40 kDa dextran across the BBB in B^3^C was analyzed offline using NIS Elements imaging software. Using our previously reported methods [11], we used the following equation to calculate permeability (P) of dextran across BBB in B^3^C:

$$P\;=\;\frac{1}{I_{v0}}\frac{V}{S}\frac{dI_{t}}{dt}$$ (1)

where *I*_*t*_ is the average intensity in the tissue compartment, *I*_*v0*_ is the maximum fluorescence intensity of the vascular channel, V/S is the ratio of vascular channel volume to its surface area.

The permeability of an empty device was subtracted from the total apparent permeability to obtain the actual permeability of RBEC under different conditions; following Eq 2 was used for this calculation.

1(P)RBEC = 1(P)Total−1(P)Cell−free (2)

For electrical resistance measurements in B^3^C, an electrode compartment was created outside the vascular channel. Barrier resistance was determined using a 2-point measurement system. Ag/AgCl electrodes were inserted into the tissue compartment and the electrode compartment and connected to SynVivo Cell Resistance Analyzer (CFD Research Corporation, Huntsville AL). These electrodes were then used to apply a constant 200 mV sinusoidal voltage signal at 1 kHz and the resulting current was used to calculate the resistance. Resistance of cell-free B^3^C (control) or B^3^C with RBEC or RBEC + ACM was measured every day for up to six days after seeding of the cells.

### Measurement of Permeability and Electrical Resistance in Transwell Model

Transwell based BBB models have been used extensively to study the BBB. Thus, we compared the performance of B^3^C to the transwell BBB model using neonatal RBEC. Transwell inserts (polyester membrane 12-well plate transwell, membrane pore size = 3 μm, membrane surface area = 1.12 cm^2^, Corning Inc., Lowell, MA) were coated with rat fibronectin (5 μg/cm^2^, EMD Millipore) overnight at 4°C. The next day, RBEC from a confluent culture were harvested and seeded at a density of 175,000–200,000 cells/well (150,000–180,000 cells/cm^2^) on the apical side of the transwell inserts using RBEC medium containing 20% BPDS [31]. After overnight incubation in the CO_2_ incubator at 37°C, the medium was replaced with fresh RBEC medium or RBEC medium containing 50% ACM. Medium in the transwells was replaced with fresh medium every 2–3 days. These cultures were maintained for 5 days before used for permeability measurements. For measuring permeability of transwell BBB, Texas-Red 40 kDa dextran (25 μM in RBEC medium) was added to the apical side of the transwell. At fixed time intervals, 50 μl of sample was collected from the basolateral side, which was replaced by 50 μl fresh RBEC medium to avoid volume dependent concentration gradients. A plate reader (Tecan Infinite 200 Pro) was used to measure the fluorescence level of collected samples from the basolateral compartment over time and to calculate its permeability using Eqs 1 and 2 above.

Electrical resistance measurements in transwell were carried out using EVOM2 TEER instrument with STX2 chopstick electrodes (World Precision Instruments, Sarasota, FL). Resistance of cell-free transwell (control) or transwell with RBEC or RBEC + ACM was measured every day for up to six days after seeding of the cells.

### Immunocytochemistry

For immunocytochemistry of tight junction protein ZO-1, astrocytic marker GFAP and microglial marker CD11b, cells were fixed in ice cold methanol at -20°C (for ZO-1 staining) or in 4% paraformaldehyde at room temperature (for GFAP and CD11b double immunostaining) for 10 min [13], permeabilized with 0.1% Triton X-100 for 10 min (only for GFAP) at room temperature and subsequently blocked with 10% goat serum in 1X PBS for one hour at room temperature. For the detection of tight junction protein, ZO-1 in RBEC, a rabbit polyclonal antibody to ZO-1 was used (1:100 dilution). For the detection of GFAP expression in astrocytes, a polyclonal rabbit antibody to GFAP was used (1:250 dilution). The fixed cells were incubated with primary antibodies at 4°C overnight followed by species-specific AlexaFluor 488 or AlexaFluor 594 secondary antibody (1:1000 dilution) for one hour at room temperature in the dark. Nuclei were counterstained using Hoechst 33342 nuclear stain in PBS at 37°C for 10–15 min. Cells were examined using a Nikon TE200 fluorescence microscope using Nikon’s NIS elements software.

For confocal imaging of filamentous-actin in RBEC, cells were fixed in 4% paraformaldehyde at room temperature for 10 min followed by permeabilization with 0.1% Triton X-100 for 10 min at room temperature. Alexa Fluor^®^ 488 Phalloidin, a high affinity filamentous (F-actin) probe conjugated to green-fluorescent Alexa Fluor^®^ 488 dye (A12379, 200 U/ml, 1:20 dilution, Life Technologies Corporation), was used to stain the RBEC cytoskeleton through the binding of phalloidin to F-actin and a fluorescent DNA dye, Draq5 (62.5 μM, 1:80 dilution) was used as the nuclear stain. Cells were allowed to incubate for 15 minutes at room temperature and then washed with PBS. A Leica SP-1 laser scanning confocal microscope was used to examine the cells. Confocal microscopy images were individually acquired from color channel and reconstructed into 3D rendering of RBEC in the vascular channels of B^3^C using Image J software (NIH, version: 1.47v).

### Measurement of Permeability across *In Vivo* BBB in Neonatal Rat

All procedures involving animals were *carried out in strict accordance with the recommendations in the Guide for the Care and Use of Laboratory Animals of the National Institutes of Health*. *The protocol was approved by the Institutional Animal Care and Use Committee of Temple University (Permit Number*: *4179)*. *All efforts were made to minimize suffering*. Sprague Dawley (SD) rats were obtained from Taconic Farms (Hudson, NY). *In vivo* BBB permeability of fluorescein isothiocyanate (FITC) conjugated 40 kDa dextran (Sigma-Aldrich) was measured in two week old SD rats using our previously described protocol [11]. Briefly, animals were anesthetized with an intraperitoneal (i.p.) injection of a solution of ketamine (87 mg/ml) and xylazine (13 mg/ml) at an appropriate dose (0.5 ml/kg body weight) and a cranial window was created as described previously [11]. The fluorescent dextran (50 mg/ ml, 1 ml /kg body weight) was injected into the jugular vein through a cannula. The intensity of the fluorescent dextran was measured (Rollera Bolt fluorescence camera from QImaging, Surrey, BC, Canada) in the vessels and brain tissue for 5 seconds every minute and was used to estimate permeability of 40 kDa dextran. At the end of the experiment, the animals were euthanized by overdose of pentobarbitol and then exsanguination.

### Statistical Analysis

A paired student’s t-test was used for comparing two means for the effect of a treatment. One-way ANOVA was used for comparing more than two means with single factor and Tukey’s multiple comparison test was used for comparing multiple means. Two-factor ANOVA was used for determining the effect of two independent factors and Tukey’s multiple comparison test was used for comparing multiple means. Statistical analyses were performed using SigmaPlot 12.3 (San Jose, CA) and p<0.05 was considered significant.

## Results

### Development of a Fluorescence Microscopy Based Method for Measuring Permeability in B^3^C

A photomask was used to construct B^3^C with two independent vascular channels with dimensions of 200 μm x 100 μm x 2762 μm (width x height x length) placed around the tissue compartment (Fig 1A). The 3D geometry of vascular channels and dimensions that closely approximate the size and morphology of microvessels *in vivo* permit the B^3^C model to maintain shear flow conditions in vascular channels. As shown in Fig 1A and 1B, the side-by-side placement of optically clear vascular channels and the tissue compartment in B^3^C permits simultaneous real-time visualization of both compartments. The porous interface allows for biochemical and cellular communication between the two compartments. As schematically shown in Fig 1B, B^3^C is designed to allow culturing of brain cells, e.g. astrocytes, in the tissue compartment and endothelial cells in vascular channels of the device. To allow a real-time monitoring of BBB function *in vitro* e.g. the assessment of barrier permeability in B^3^C, the device is constructed from optically clear PDMS assembled on a microscope slide as shown in Fig 1C.

**Fig 2:**
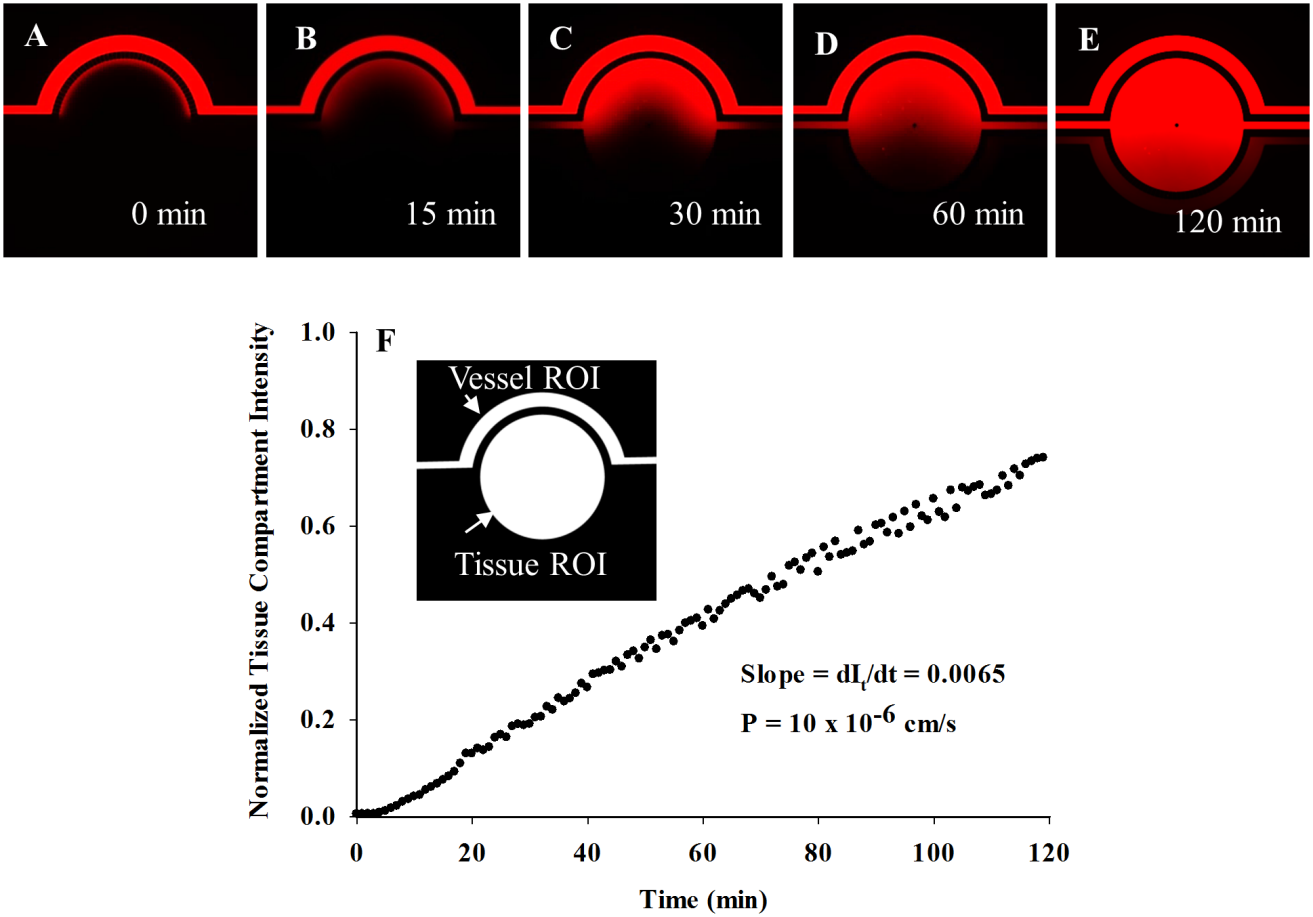
Passage of fluorescent dextran from the vascular channel to tissue compartment of B^3^C under shear flow. Permeability of Texas Red 40 kDa dextran from vascular channel to the tissue compartment in a cell-free B^3^C after 5 min (A), 15 min (B), 30 min (C), 60 min (D) and 120 min (E) from the initiation of flow in vascular channel. Normalized tissue intensity in a cell-free B^3^C increases linearly with time in the tissue compartment (F).

Since the B^3^C developed here is a dynamic *in vitro* model of neonatal BBB suitable for real-time monitoring and direct measurement of permeability across BBB using microscopic methods, we first optimized the techniques for quantifying permeability in B^3^C. Permeation of the fluorescent 40 kDa dextran from the vascular channel to the tissue compartment of cell-free B^3^C was characterized by imaging over time as dextran was injected into the vascular channel at a flow rate of 0.2 μl/min (i.e. shear stress of 7.6x10^-2^ dynes/cm^2^). As shown in Fig 2A, 2B, 2C, 2D and 2E, fluorescent dextran accumulates in the tissue compartment in a time-dependent manner over a 120 min period. Quantification of permeability was performed by calculating the average intensity of fluorescent dextran in the entire tissue compartment and normalizing it to the maximum intensity of fluorescent dextran in the vascular channel. The results of a typical cell-free experiment shown in Fig 2F indicate that the normalized intensity increases linearly with time in the tissue compartment. The slope of the line (dI_t_/dt) in Fig 2F is used to calculate the permeability of dextran from the vascular channel to the tissue compartment using Eq 1, which gives (P)_Cell-Free_ (in this case 10 x 10^−6^ cm/s) used in Eq 2.

### Neonatal RBEC form a Complete Lumen along the Walls of the Vascular Channel and Exhibit Increased Expression of Tight Junctions in B^3^C

**Fig 3:**
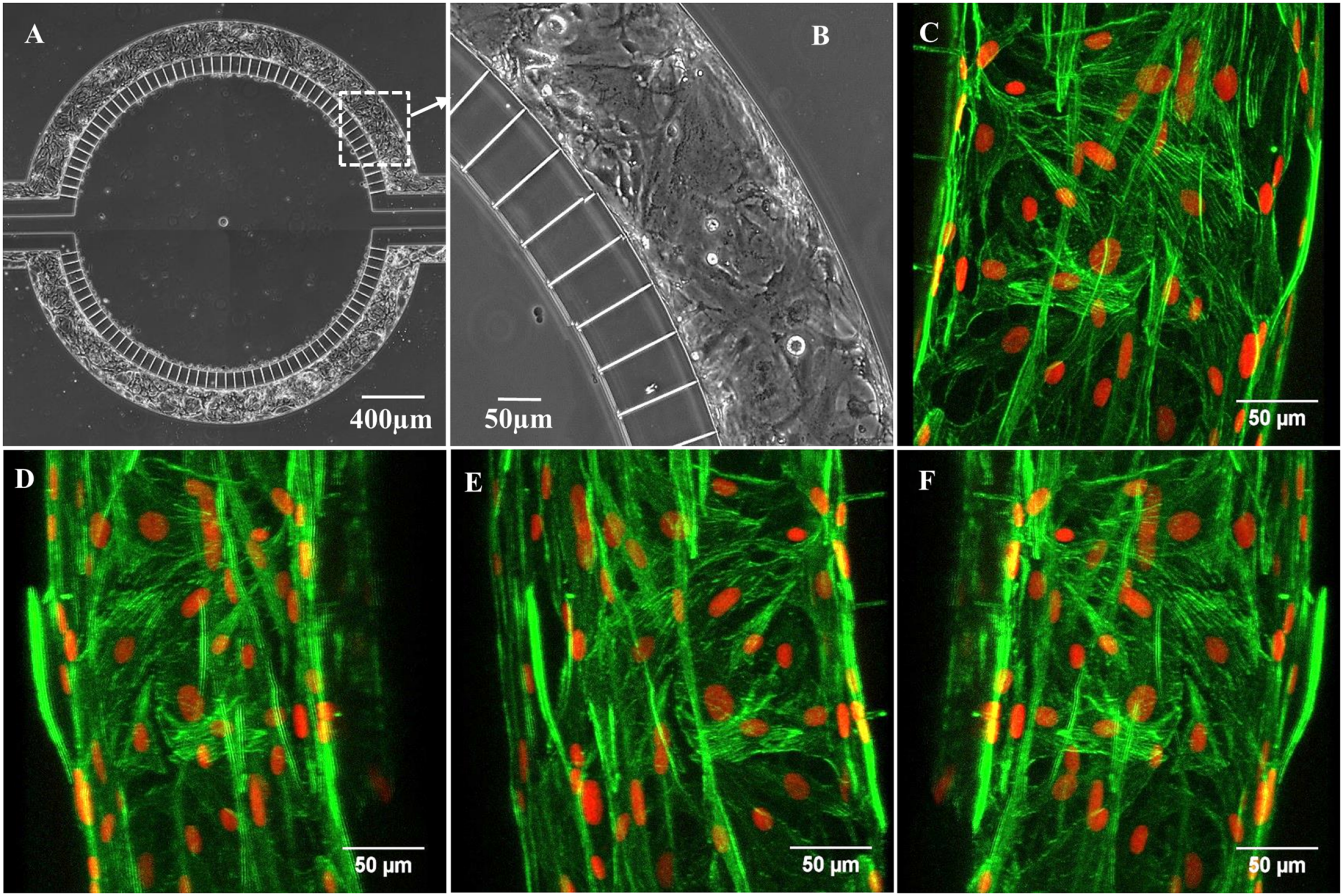
Neonatal RBEC cultured under flow in the vascular channel of B^3^C form a complete lumen. Full view of B^3^C device showing RBEC cultured in the two vascular channels (A). Magnified view of inset from panel (A) showing a section of the vascular channel with cultured RBEC (B). 3D reconstruction of confocal images of neonatal RBEC cultured in B^3^C stained with f-actin (green) and Draq5 (red) after 5 days in culture maintained under flow of 0.01 μl/min in RBEC medium (C)—(F); images are shown with a Y-axis rotation of 0, 140, 210 and 330 degrees in (C), (D), (E) and (F) respectively. All images were acquired after 5 days of 0.01 μl/min of flow of RBEC medium over cultured RBEC in the vascular channel of B^3^C.

RBEC (from passage 3) were seeded on a fibronection coated inner surface of vascular channels of B^3^C and maintained under a flow rate of 0.01 μl/min (i.e. shear stress of 3.8x10^−3^ dynes/cm^2^) in RBEC culture medium in 5% CO_2_/95% air at 37°C for 5 days. As shown in Fig 3A and 3B, RBEC growing in the vascular channel of B^3^C exhibit the morphology of healthy, confluent endothelial cells after 5 days of culture (additional images of cell culture in B^3^C over time are shown in S1 and S2 Figs). The formation of the lining by RBEC on the inner surface of the vascular channels of B^3^C was examined by confocal microscopy by fluorescent staining of the filamentous-actin (f-actin) to visualize the actin cytoskeleton and fluorescent staining of the nuclei with Draq5. As shown in Fig 3B through 3F, after 5 days in culture the vascular channels were lined by a continuous sheet of endothelial cells forming a complete lumen mimicking the *in vivo* tubular morphology of microvessels.

**Fig 4:**
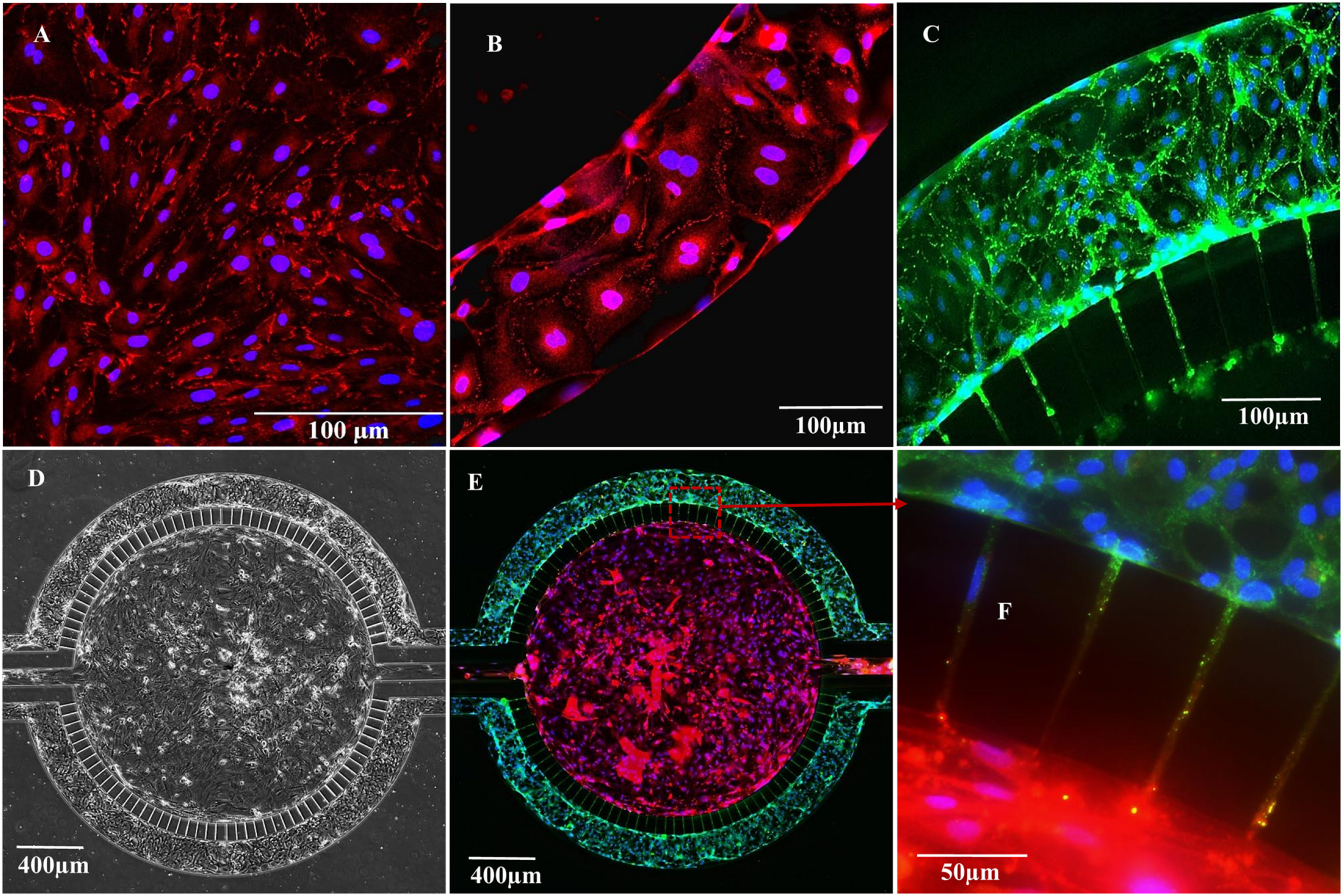
Tight junction formation by neonatal RBEC under static and flow conditions as indicated by immunofluorescence staining of ZO-1. RBEC cultured for 5 days under static conditions were stained with ZO-1 (red) and Hoechst 33342 (blue) (A). RBEC cultured for 5 days under flow of RBEC medium in B^3^C were stained with ZO-1 (red) and Hoechst 33342 (blue) (B). RBEC cultured under flow of ACM for 5 days in B^3^C were stained with ZO-1 (green) and Hoechst 33342 (blue) (C). Bright field image of B^3^C showing RBEC in the vascular channels after 5 days of co-culture with rat astrocytes in the tissue compartment of B^3^C (D). Immunofluorescence staining of RBEC in vascular channel for ZO-1 (green) and astrocytes in tissue compartment for GFAP (red) (E). Magnified view of interface with pores from panel E showing staining of cells inside the pores, ZO-1 (green), GFAP (red) and nuclei (blue) (F).

Endothelial cells exhibit barrier formation by expressing tight junction proteins (e.g. ZO-1) that are an integral part of the tight junction complexes at the cell-cell contact that make up the BBB [27]. The ZO-1 signal was detected at intercellular junctions 5 days after the initiation of culture maintained under static conditions (Fig 4A). Cultures of RBEC maintained under static conditions exhibited discontinues and/or patchy loss of tight junctions, as assessed by ZO-1 staining. By contrast, ZO-1 expression was more uniform and continuous when RBEC were cultured under flow conditions in B^3^C (Fig 4B). Furthermore, the ZO-1 expression was uniform and markedly enhanced when RBEC were cultured under flow in the presence of ACM as shown in Fig 4C. Another important characteristic of a functional *in vitro* BBB is the ability of the astrocytes to exert brain (i.e. astrocytic) function on the endothelium. Thus, we co-cultured rat astrocytes (from passage 2) with RBEC (from passage 3) in our B^3^C model, the result of which are shown in Fig 4D through 4F. Fig 4D shows the bright field image of the co-culture of RBEC under shear flow in the vascular channel and astrocytes in the tissue compartment under static conditions. Fig 4E shows the immunofluorescence staining of RBEC and astrocytes in the co-culture B^3^C model. Similar to RBEC cultured in the presence of ACM (Fig 4C), stronger expression of ZO-1 was observed at the intercellular junctions of RBEC when co-cultured with astrocytes (Fig 4E). To confirm the presence of GFAP expression in rat neonatal astrocytes grown in the tissue compartment of B^3^C we stained these cells with antibody against GFAP five days following seeding (Fig 4E). RBEC and astrocyte nuclei were counterstained with Hoechst 33342 nuclear stain. Fig 4F shows magnified view of the interface with pores and a substantial red (GFAP), and green (ZO-1) with occasional blue (nuclei) staining detected inside the 3 μm pores along the interface between vascular channels and tissue compartment. Cultured cells exhibit long cytoplasmic processes that allow contact between the astrocytes and endothelial cells through the porous interface, with only a few pores showing RBEC migration from the vascular channel to the tissue compartment (S3 Fig). This shows that our neonatal B^3^C model not only provides an *in vivo*-like shear flow environment, but also permits communication of astrocytes cultured in the tissue compartment with endothelial cells cultured in the vascular channels by direct physical endfeet-like contact. Altogether, these results demonstrate that shear flow, presence of astrocytes or the factors secreted by astrocytes modulate tight junction formation in B^3^C model, as monitored by the expression of endothelial tight junction protein ZO-1 at the margins of cell contact, indicating that our B^3^C model resembles *in vivo* conditions.

### ACM or Co-culture of RBEC with Astrocytes Significantly Improves Barrier Properties of Neonatal B^3^C

**Fig 5:**
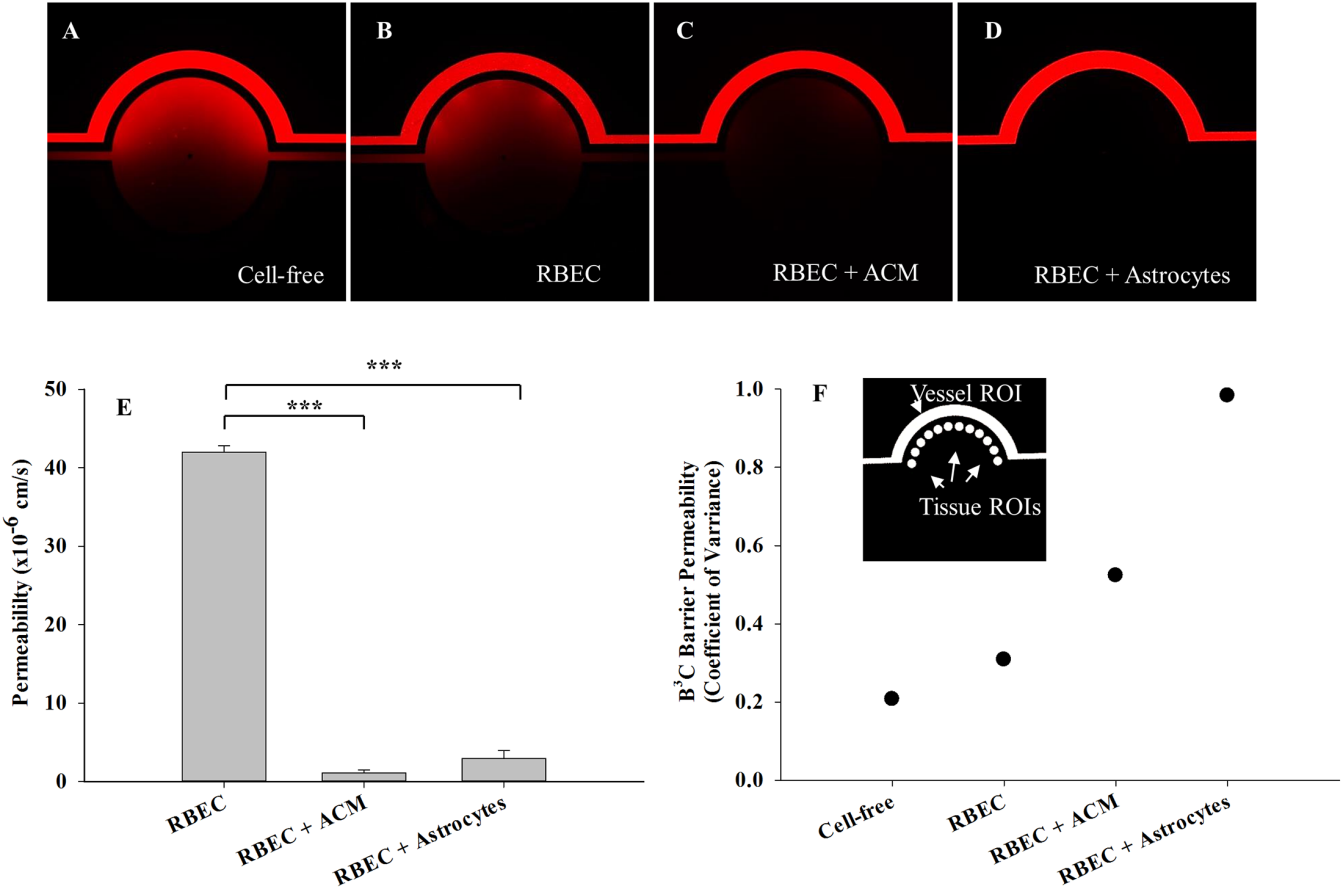
Real-time analysis of passage of fluorescent dextran from the vascular to tissue compartment of B^3^C under shear flow. Compared to cell-free B^3^C (A) or RBEC alone (B), presence of ACM (C) and co-culture with rat astrocytes (D) improves barrier function of neonatal RBEC in B^3^C as detected by the passage of Texas Red 40 kDa dextran from the vascular channel to the tissue compartment of B^3^C under shear flow. The passage of Texas Red 40 kDa dextran was monitored and imaged by Fluorescent microscopy (Nikon TE200). Representative images acquired 60 min after the initiation of flow in the vascular channel (A-D). Quantification of permeability shows that RBEC cultured in the presence of ACM or RBEC co-cultured with astrocytes exhibit significant reduction in the permeability of 40 kDa dextran compared to RBEC alone (E). Coefficient of variance of measured intensities of fluorescent dextran at 12 regularly spaced ROIs in the tissue compartment immediately adjacent to the vascular channel at 60 min after the initiation of the flow was used as an index of permeability heterogeneity. Variation in permeability is lowest in cell-free B^3^C but increases as the microenvironment of B^3^C becomes more realistic (F). (*** indicates significant difference p<0.001, one-way ANOVA with Tukey’s multiple comparison test, n = 3–4 experiments per group).

Barrier formation by the brain endothelium is facilitated by the presence of glial cells, such as astrocytes, and the factors secreted by astrocytes [19, 20]. In addition to assessing tight junction formation as described above, permeability of tracers such as dextran can be used to quantify the tightness of the barrier formed by RBEC in B^3^C model. Using the B^3^C model we observed that barrier permeability was dependent on the presence of astrocytes or ACM. Significant reduction of passage of Texas Red 40 kDa dextran from the vascular channel to tissue compartment was observed when RBEC (from passage 3) were cultured under flow (0.01 μl/min, i.e. shear stress of 3.8x10^−3^ dynes/cm^2^) in the vascular channels in the presence of ACM or astrocytes (from passage 2) cultured in tissue compartment (Fig 5C and 5D, respectively) as compared to RBEC alone (Fig 5B) or cell-free device (Fig 5A). Flow rate used for permeability experiments was 0.2 μl/min (i.e. shear stress of 7.6x10^-2^ dynes/cm^2^). As shown in Fig 5E, permeability of 40 kDa dextran in B^3^C cultured with RBC alone (41.0±0.9 x 10^−6^ cm/s) was significantly reduced when RBC were cultured in the presence of ACM (1.1±0.4 x 10^−6^ cm/s) and when RBC were co-cultured with astrocytes (2.9±1.0 x 10^−6^ cm/s).

In the vasculature of the brain and other tissues *in vivo*, permeability is reported to be heterogeneous along the length of a vessel [35–37]. Coefficient of variance for the measured intensities of fluorescent dextran at 12 regularly spaced regions of interest (ROIs) in the tissue compartment immediately adjacent to the vascular channel at 60 min after the initiation of the flow was used to assess the heterogeneity of permeability along the vascular channel in B^3^C (Fig 5F). As expected, variability in permeation was lowest in cell-free B^3^C due to the uniform structure of the wall of the vascular channels. However, while the overall permeability of B^3^C decreases with the inclusion of ACM or astrocytes in its microenvironment (Fig 5E), the extravasation of dextran becomes more heterogeneous along the vascular channel (Fig 5F).

### B^3^C Exhibits Significantly Improved Barrier Properties Compared to Transwell and Closely Mimics the *In Vivo* Neonatal BBB

**Fig 6:**
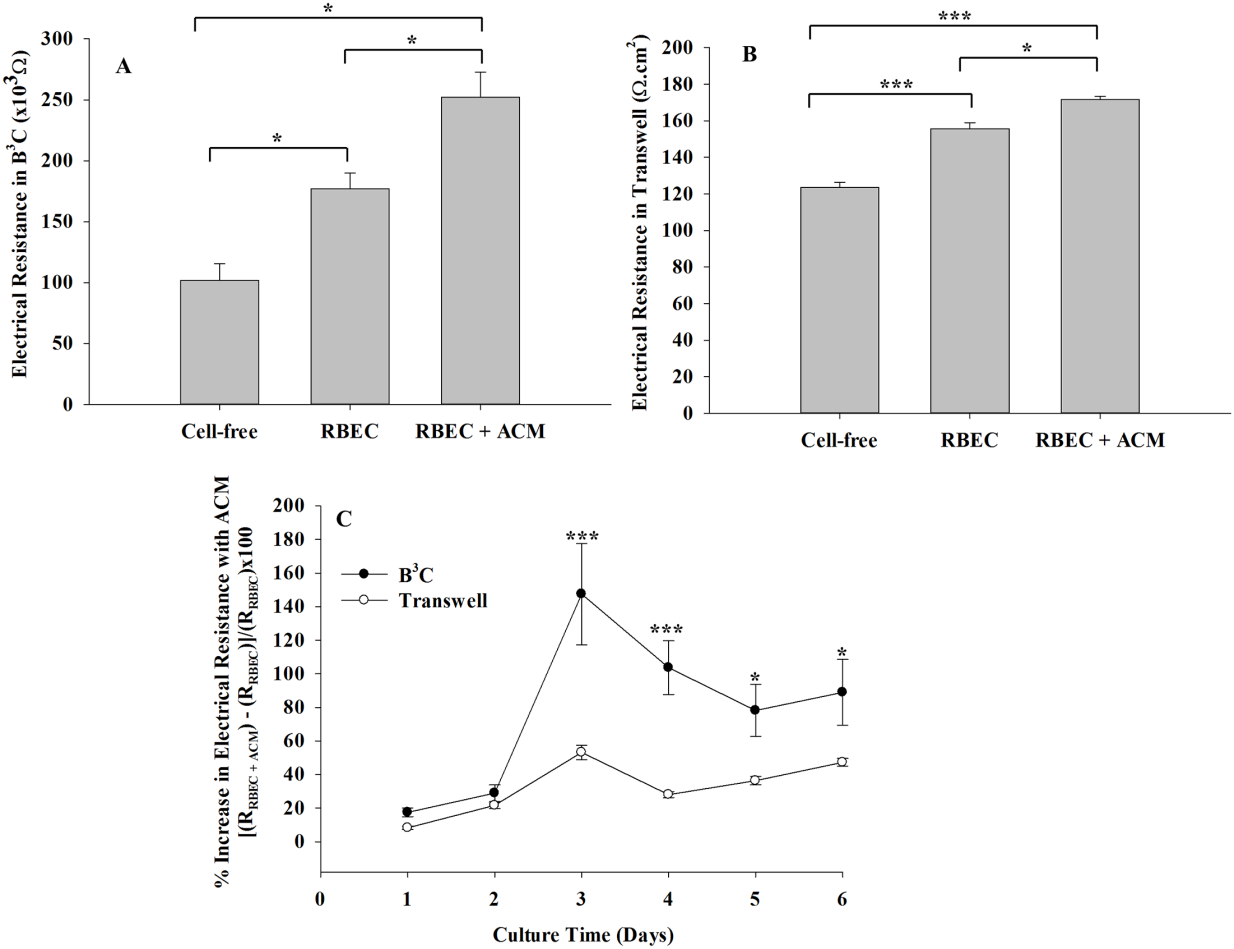
ACM enhances the barrier properties of neonatal RBEC more significantly in B^3^C as compared to transwell. Presence of ACM increases electrical resistance of neonatal RBEC in both B^3^C (A) and transwell (B), the electrical resistance measurements are from day 5. Presence of ACM increases resistance more significantly in B^3^C as compared to the transwell model (C). Please note that the units of electrical resistance for B^3^C and transwell are different as noted in the results section. [* indicates significant difference p<0.05, ** indicates significant difference p<0.01, *** indicates significant difference p<0.001, one-way ANOVA (panels A and B) or two-way ANOVA (panel C) with Tukey’s multiple comparison test; n = 3 experiments per group].

To further assess the functional characteristics of the neonatal BBB, we measured electrical resistance of neonatal RBEC (from passage 3) cultured in the presence and absence of ACM in B^3^C and transwell. As shown in Fig 6A and 6B and S1 Table, neonatal RBEC form a tighter barrier when cultured in the presence of ACM as indicated by a significant increase in the electrical resistance in both B^3^C and transwell. Note that the pore design in B^3^C and the methodology and instrumentation used for measuring electrical resistance in B^3^C are novel and significantly different from that of transwell. For example, surface area and the pore densities are significantly different between B^3^C and transwell. Thus, the electrical resistance of B^3^C (reported in Ω) cannot be directly compared with electrical resistance in transwell (reported in Ω-cm^2^). The effect of ACM on RBEC barrier formation in these two systems were compared by calculating percent increase in their respective electrical resistances. Our findings indicate that the ACM causes significantly higher increase in the electrical resistance of neonatal RBEC in B^3^C compared to transwell, thus forming a tighter barrier in B^3^C (Fig 6C).

**Fig 7:**
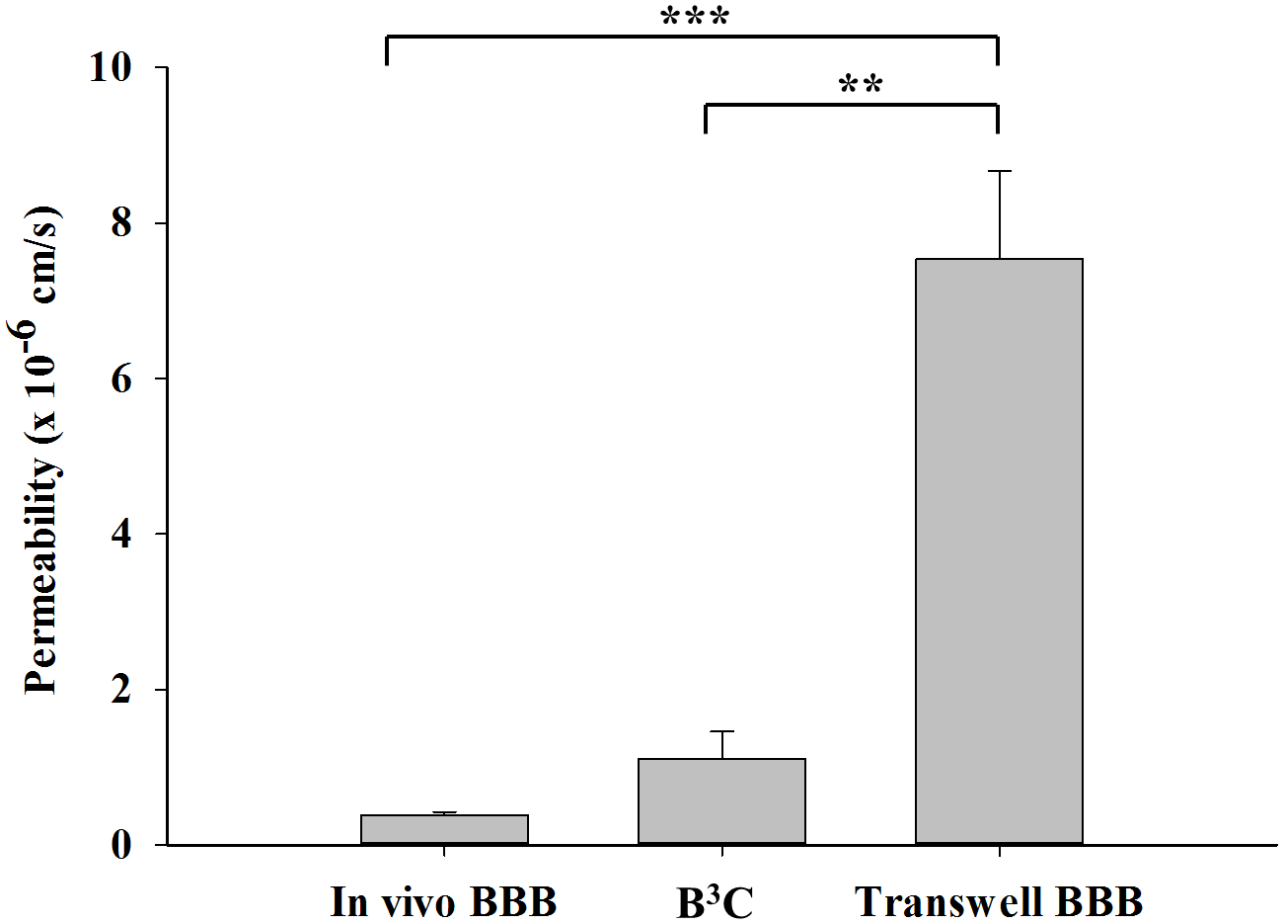
B^3^C exhibits significantly improved barrier function compared to the transwell model and closely approximates the permeability of neonatal *in vivo* BBB. B^3^C and Transwell BBB were constructed with neonatal RBEC in the presence of ACM. Permeability of 40 kDa dextran in B^3^C is significantly lower than transwell but not significantly different from that of *in vivo* BBB in neonatal rats. (** indicates significant difference p<0.01, *** indicates significant difference p<0.001, one-way ANOVA with Tukey’s multiple comparison test; n = 3–4 experiments per group).

Consistent with our observations in B^3^C, the permeability of 40 kDa dextran in transwell model decreases from 13.8±0.8 x 10^−6^ cm/s when RBEC were cultured in the absence of ACM to 7.5±1.1 x 10^−6^ cm/s when RBEC were cultured in the presence of ACM, confirming that the presence of astrocytic factors is essential for forming a tighter blood-brain barrier. Nevertheless, as shown in Fig 7, permeability of 40 kDa dextran in B^3^C (1.1±0.4 x 10^−6^ cm/s) is significantly lower than transwell (7.5±1.1 x 10^−6^ cm/s) but not significantly different from that of *in vivo* BBB in neonatal rats (0.4±0.05 x 10^−6^ cm/s). Combined, these results indicate that the microenvironment in B^3^C closely approximates the neonatal *in vivo* BBB.

**Fig 8:**
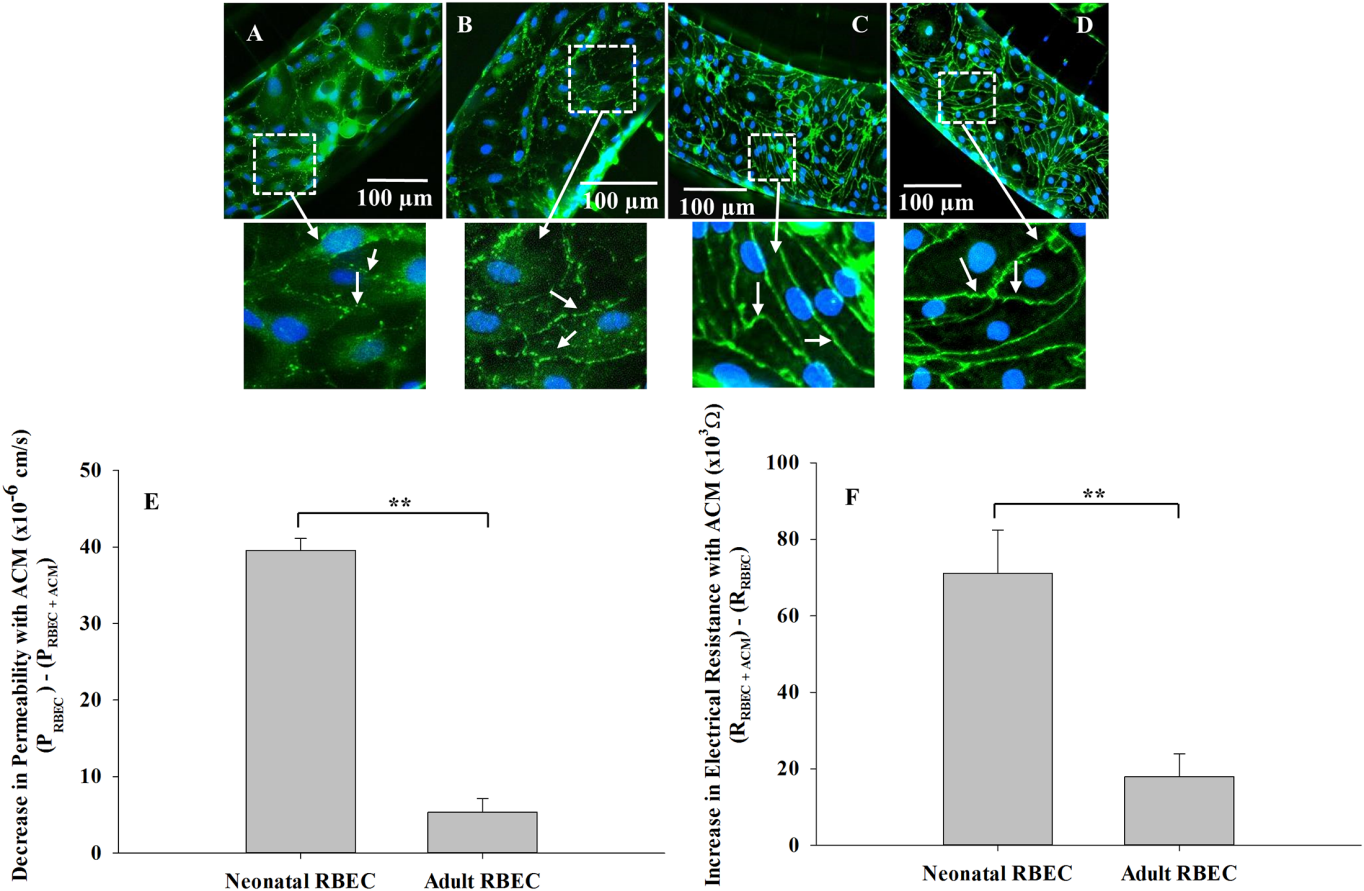
Neonatal RBEC exhibit distinct barrier structure and function compared to adult RBEC in B^3^C. Neonatal RBEC alone (A) and neonatal RBEC + ACM (B) exhibit distinctly weaker and less organized ZO-1 staining (arrows) compared to adult RBEC alone (C) and adult RBEC + ACM (D). In the presence of ACM, neonatal RBEC exhibit discontinuous bands of ZO-1 staining (B) compared to neonatal RBEC in the absence of ACM where ZO-1 staining is discontinuous and granular (A). Presence of ACM has a significantly larger impact on both permeability (E) and electrical resistance (F) in neonatal RBEC compared to adult RBEC. Inset panels show higher magnification of white squared regions. (** indicates significant difference p<0.01, student’s t-test; n = 3–4 experiments per group).

In this study, we used RBEC isolated from neonatal rats to develop the novel neonatal BBB on a chip. To establish that this model indeed exhibits BBB structural and functional characteristics that are different from adult BBB, we also constructed B^3^C using RBEC from adult Sprague Dawley rats (from passage 4) and the barrier structure and function of the BBB formed by neonatal and adult RBEC in B^3^C were compared. As shown in Fig 8, neonatal RBEC alone (Fig 8A) and in the presence of ACM (Fig 8B) exhibit weaker and less organized ZO-1 staining compared to both adult RBEC alone (Fig 8C) and adult RBEC in the presence of ACM (Fig 8D). Consistent with results presented in Figs 5 and 6, culturing neonatal RBEC with ACM in B^3^C improves barrier properties as indicated by the stronger ZO-1 staining (discontinuous weak granular ZO-1 staining in Fig 8A compared to discontinuous but stronger band-like ZO-1 staining in Fig 8B). On the other hand, culturing adult RBEC with ACM in B^3^C does not significantly improve barrier properties as indicated by ZO-1 staining (similar continuous ZO-1 staining in both Fig 8C and 8D). Furthermore, presence of ACM in B^3^C decreases permeability of 40 kDa dextran (Fig 8E) and increases electrical resistance (Fig 8F) more significantly in neonatal RBEC as compared to adult RBEC.

## Discussion

*In vitro* BBB models that closely mimic the in *vivo* BBB microenvironment are valuable tools for the study of neonatal BBB function as well as for screening of novel therapeutics. Transwell BBB models approximate several of the important aspects of the *in vivo* BBB and are routinely used for BBB studies and for screening neurotherapeutics [38, 39]. More recently, dynamic flow-based *in vitro* BBB models have also been developed to better reproduce the *in vivo* microvascular environment [17, 18, 40, 41]. However, existing *in vitro* BBB models have a number of important limitations and for the most part do not mimic the microenvironment of the neonatal BBB. For example, while the DIV-BBB and NDIV-BBB models developed by Cucullo et al. represent a significantly improved design of *in vitro* BBB models, they still do not accurately represent the brain microvascular dimensions and geometry [17, 18, 40, 41]. Furthermore, development of suitable *in vitro* BBB models of neonatal BBB is particularly important since there are significant differences in structure and function of neonatal and adult BBB [7, 26–28]. Having a relevant *in vitro* model of neonatal BBB is important for understanding neonatal neural pathogenesis and for developing appropriate treatment strategies.

This study demonstrates, for the first time, an *in vitro* neonatal BBB model that shows a significant improvement from the traditional transwell model and is able to mimic the *in vivo* brain microenvironmental conditions (e.g. shear flow and presence of glial cells). In order to develop and characterize an *in vitro* model of neonatal BBB, we designed a neonatal BBB on a chip (B^3^C) model for the co-culture of primary neonatal brain endothelial cells and neonatal astrocytes. In contrast to other microfluidic systems, the B^3^C design includes a tissue compartment enclosed by vascular channels. The tissue compartment and vascular channels are in communication via an interface with 3 μm pores along the length of the vascular channels, replacing the use of membranes in conventional BBB models. The three-dimensional geometry of vascular channels in B^3^C addresses the important challenge of developing *in vitro* BBB models with shear flow conditions which have been shown to be critical in the formation of realistically tight barriers [17, 21]. Furthermore, the optically clear microfluidic chip, and the architecture of the device, allows for visualization and real-time measurements of the dynamic interactions occurring in vascular channels and tissue compartment.

As shown in Fig 3, neonatal rat brain microvascular endothelial cells (RBEC), grown on the fibronectin coated inner surfaces of vascular channels of the B^3^C form the endothelial lining along vascular channels with a complete lumen, thus mimicking the tubular morphology of the *in vivo* microvessels. The ability to reproduce the tubular morphology of the *in vivo* microvessels represents a significant advancement in our efforts to model the *in vivo* BBB. Furthermore, the ability to visualize the dynamic processes in the neonatal B^3^C model in real time is a significant advantage as compared to flow based hollow fiber dynamic BBB models and other membrane based microfluidic approaches [17, 18, 24, 25, 40, 41]. Thus, B^3^C offers a far more realistic representation of the *in vivo* BBB microenvironment compared to currently available *in vitro* BBB models [17–23, 40, 41]. In addition to allowing for real-time analysis in an *in vivo* like microenvironment, the B^3^C has the advantage of using significantly less of the often required expensive reagents compared to the currently available *in vitro* BBB models.

The endothelium of the *in vivo* BBB is continuously exposed to both physical stimuli (e.g. shear stress) and cellular signals (e.g. presence of perivascular cells). Certain features of the BBB, for example the size and geometry of the microvessels in the brain, define the shear stress and flow patterns occurring at the *in vivo* BBB and contribute to the biochemical and functional characteristics of the BBB [1, 17, 21]. Thus, for a truly functional and physiologically realistic *in vitro* BBB, it is essential to incorporate the *in vivo* flow characteristics in the design of an *in vitro* BBB model. Accordingly, the neonatal B^3^C model realistically reproduces *in vivo* shear forces experienced by endothelial cells. We have shown that exposure of RBEC to shear forces in B^3^C improves their barrier characteristics as indicated by an increase in the expression of tight junction molecule ZO-1 (Fig 4B) compared to RBEC cultured under static conditions (Fig 4A).

Moreover, the neonatal B^3^C model was developed with a co-culture of astrocytes, which are known to contribute to the regulation and maintenance of BBB homeostasis, integrity and function [1, 18, 42–47]. We observed the functional improvement of the BBB in the neonatal B^3^C model as evidenced by the increased expression of ZO-1 (Figs 4C, 4E, 8A and 8B) and increased electrical resistance (Figs 6A, 6C and 8F) by RBEC cultured under flow conditions in the presence of ACM or astrocytes as well as by decreased permeability of the fluorescent 40 kDa dextran (Figs 5 and 8E). Nevertheless, additional studies evaluating the expression of other barrier tightening proteins such as occludin and claudin-5 may be required to fully characterize the barrier structure in B^3^C. Furthermore, our findings indicate that in B^3^C the barrier properties of neonatal RBEC are significantly different from that of adult RBEC. We also observed astrocyte protrusions and/or endfeet (indicated by the GFAP and nuclear staining in Fig 4F) through the 3 μm porous interface between the tissue and vascular compartments suggesting that the design of B^3^C allows physical and biochemical communication between the RBEC cultured in the vascular channel and the astrocytes cultured in the tissue compartment, which is essential for the formation of tighter BBB. Previously, a double monolayer formation of endothelial cells was observed in large pore sized (3 μm and upwards) transwell inserts due to endothelial cell migration through the insert pores [48]. In B^3^C, we occasionally observe that a few endothelial cells can cross the pores only during the first 1–2 days of culture (S1 Fig), but this is generally prevented by the physical hindrance of the longer migration path in B^3^C. Once the endothelial cells reach confluence, no additional migration of endothelial cells through the pores is observed. Thus, the extent of this migration is significantly lower compared to that seen by Wuest et al. (2013) for the transwell membranes and the endothelial cells do not form an extra monolayer on the other side of the vascular channel in B^3^C. Furthermore, with the presence of astrocytes in the tissue compartment of B^3^C, the endothelial migration from vascular channel to the tissue compartment is further reduced as can be seen in Fig 4E and 4F (also S2 Fig).

Growing evidence suggests that flow based *in vitro* BBB models exhibit improved performance and better approximate the *in vivo* BBB as compared to transwell based BBB models [17, 18, 21–23, 40, 41]. Consistent with these observations, the permeability of our microfluidic neonatal blood-brain barrier (B^3^C) model was found to be significantly lower than that of the transwell BBB model (Fig 7). Furthermore, as shown in Fig 7, the permeability of B^3^C model approximates the permeability of neonatal rat BBB *in vivo* supporting the notion that this system mimics many structural and functional characteristics of the *in vivo* BBB system.

In summary, we developed a dynamic neonatal BBB on a chip (B^3^C) model incorporating co-culture of neonatal rat brain endothelial cells and astrocytes. The design of B^3^C not only allows for culturing of neonatal brain endothelial cells under shear flow in three-dimensional vascular channels that mimic the dimensions of microvessels *in vivo*, but also permits interactions between the endothelial cells and brain cells. The side-by-side placement of vascular channels and the tissue compartment in optically clear PDMS on glass chip allows for real-time direct monitoring of the dynamic processes taking place in B^3^C. The microfluidic based neonatal BBB on a chip model developed in this study is a new class of *in vitro* BBB models that closely reproduces the properties of the *in vivo* BBB and delivers enhanced performance as compared with traditional transwell system.

## Supporting Information

S1 Fig Progression of RBEC culture in B^3^C. RBEC culture in B^3^C over time (A-E). Inset from (E) shows magnified view of RBEC culture at day 6 (F). In F, an occasional RBEC migrated from the vascular channel to tissue compartment is shown by an arrow; RBECs do not form a second monolayer on the other (tissue compartment) side of the porous interface.
(TIF)

S2 Fig Progression of RBEC and astrocyte co-culture in B^3^C. (TIF)

S3 Fig Astrocyte and RBEC interactions at the porous interface between vascular channel and the tissue compartment. All pores exhibit interaction between astrocytes (red GFAP staining) and endothelial cells (green ZO-1 staining). Only a few pores show some RBEC migration from the vascular channel to the tissue compartment (blue nuclear staining seen inside the pores).
(TIF)

S4 Fig Purity of astrocytes. Astrocytes shown in bright field (A) were double-stained for GFAP (B) and CD11b (C) exhibit positive staining for astrocytic marker GFAP and negative for microglial marker CD11b. Nuclear counterstaining is shown in (D) and the four channels from A-D were merged as shown in (E). Scale bar: 100 μm.
(TIF)

S1 Table Raw values of electrical resistance for B^3^C and transwell on the day of permeability measurement (post culture time of 5 days). (DOCX)
