# Supplementary figures and images for: A Novel Dynamic Neonatal Blood-Brain Barrier on a Chip
Source: PLoS One. 2015 Nov 10;10(11):e0142725. doi: 10.1371/journal.pone.0142725 (PMC4640840; doi:10.1371/journal.pone.0142725)

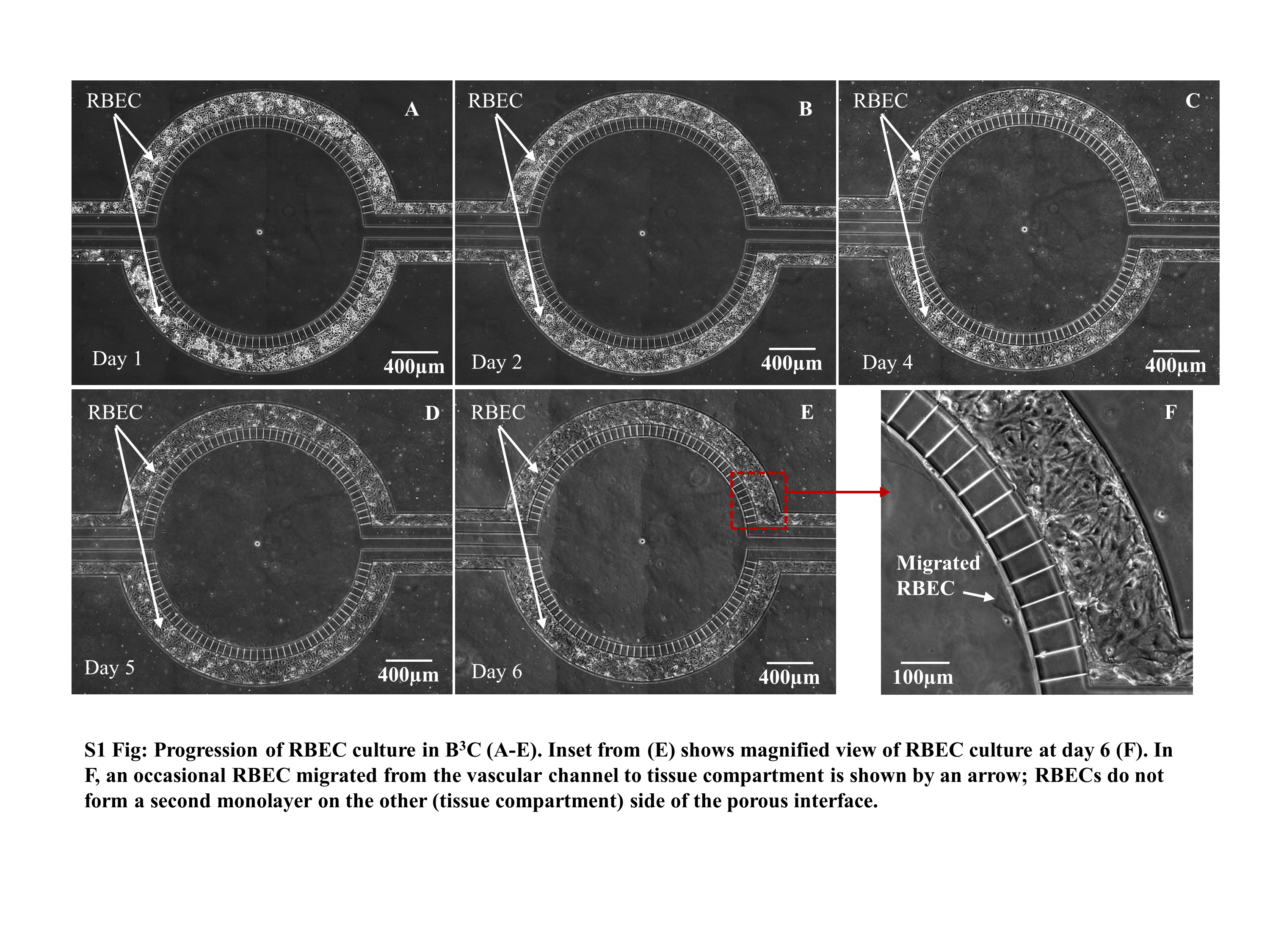

Supplement: S1 Fig — RBEC culture in B3C over time (A-E). Inset from (E) shows magnified view of RBEC culture at day 6 (F). In F, an occasional RBEC migrated from the vascular channel to tissue compartment is shown by an arrow; RBECs do not form a second monolayer on the other (tissue compartment) side of the porous interface. (TIF) [file pone.0142725.s001.TIF]

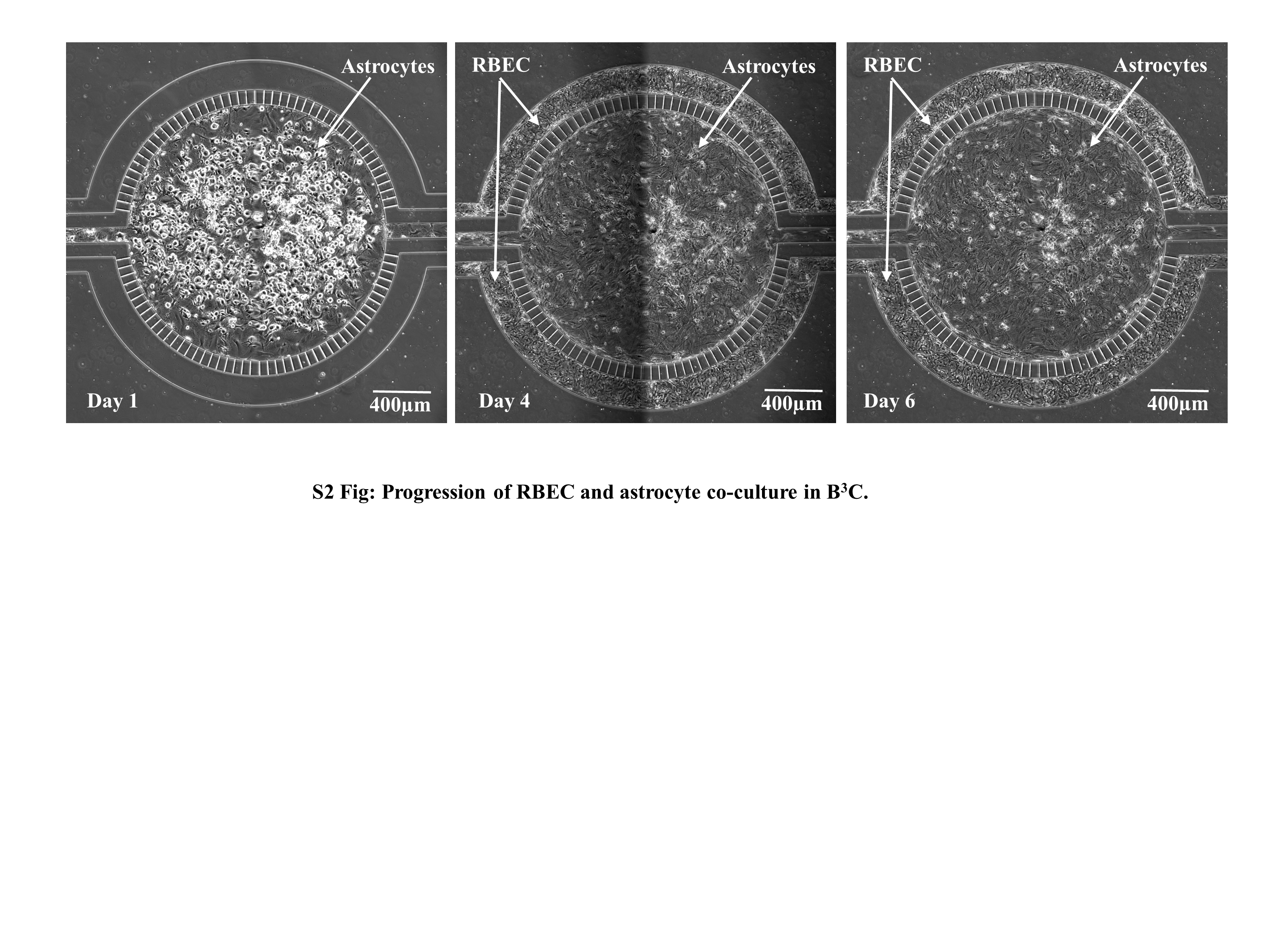

Supplement: S2 Fig — (TIF) [file pone.0142725.s002.TIF]

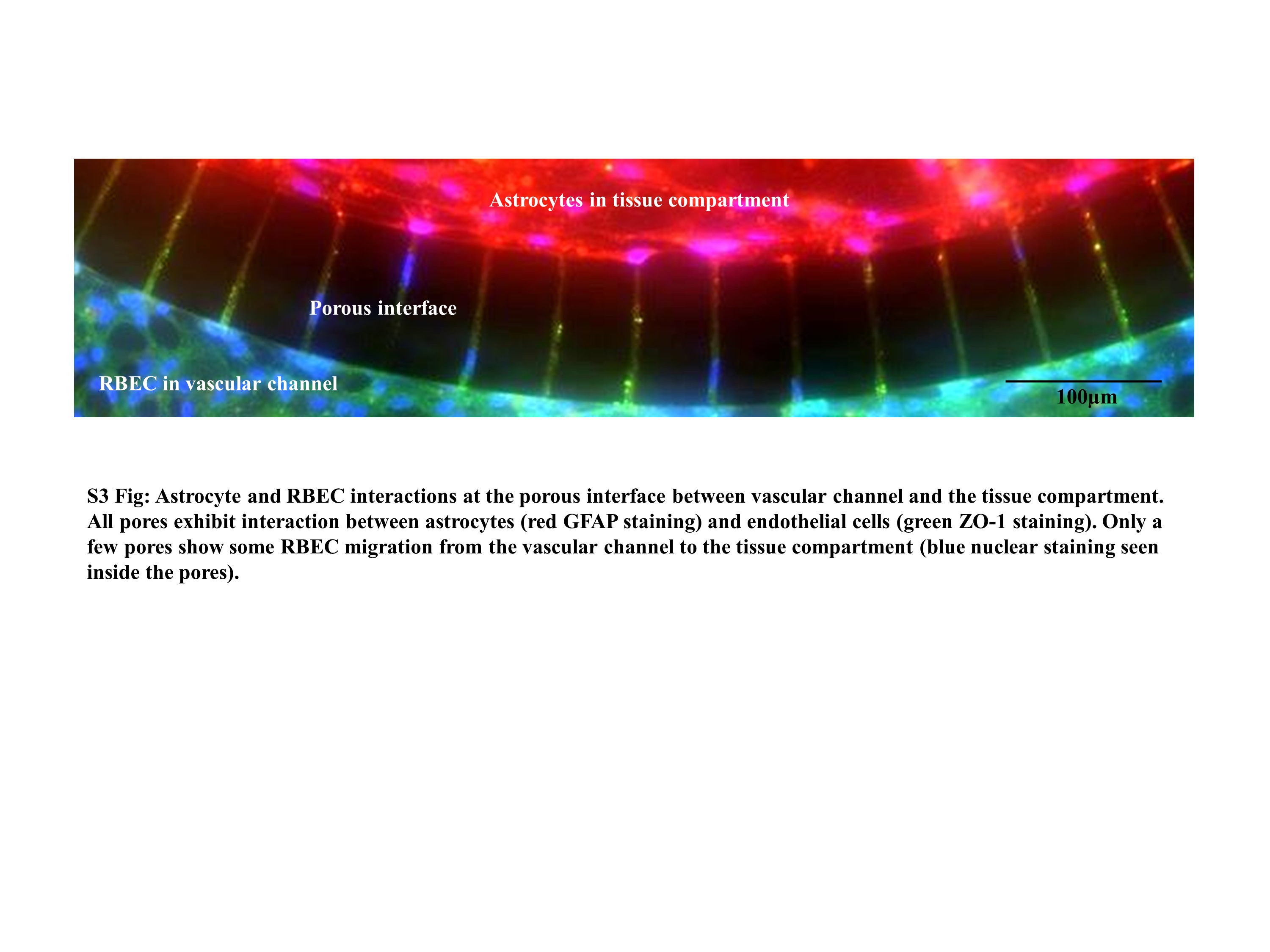

Supplement: S3 Fig — All pores exhibit interaction between astrocytes (red GFAP staining) and endothelial cells (green ZO-1 staining). Only a few pores show some RBEC migration from the vascular channel to the tissue compartment (blue nuclear staining seen inside the pores). (TIF) [file pone.0142725.s003.TIF]

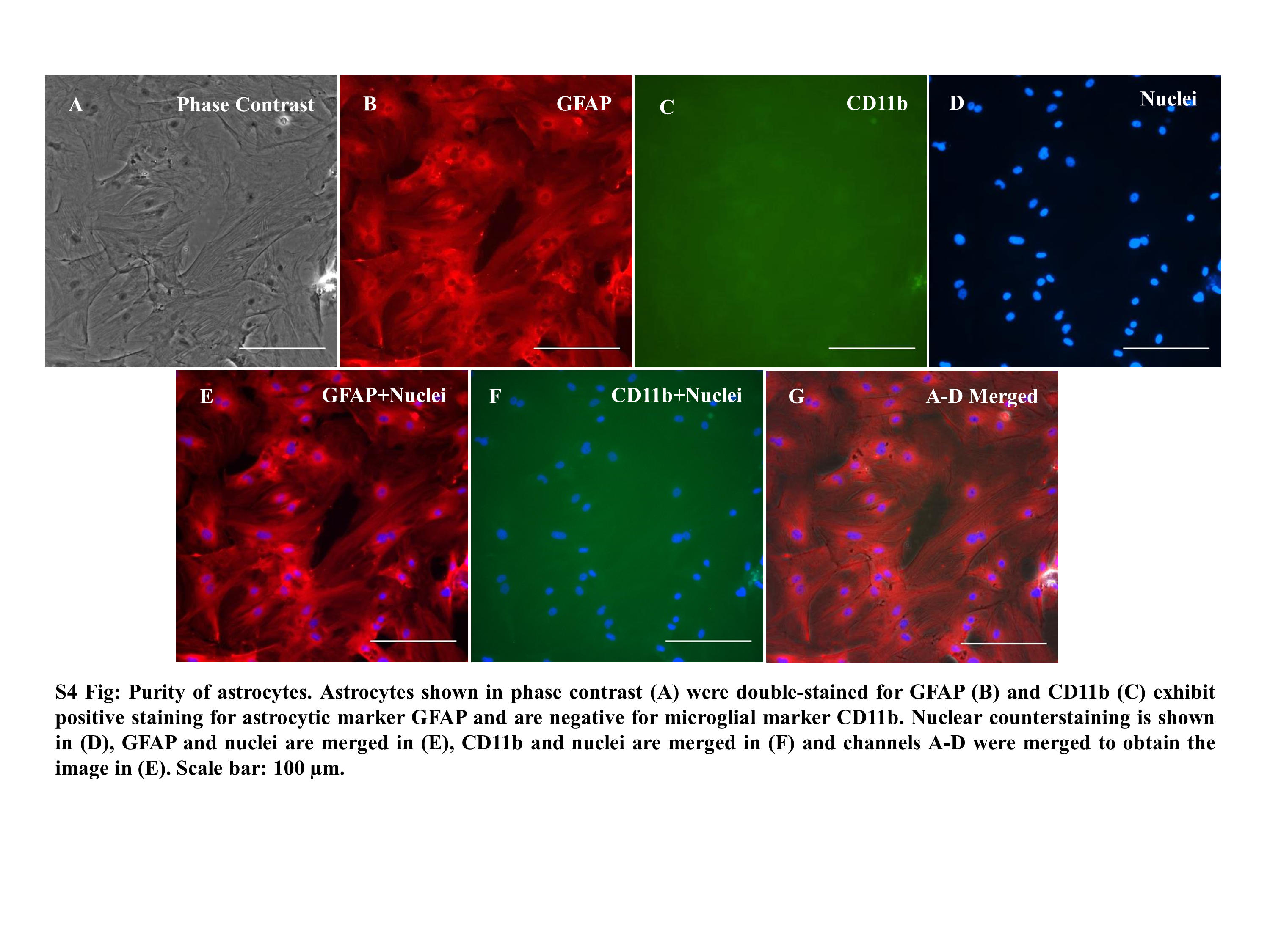

Supplement: S4 Fig — Astrocytes shown in bright field (A) were double-stained for GFAP (B) and CD11b (C) exhibit positive staining for astrocytic marker GFAP and negative for microglial marker CD11b. Nuclear counterstaining is shown in (D) and the four channels from A-D were merged as shown in (E). Scale bar: 100 μm. (TIF) [file pone.0142725.s004.TIF]
