# Supplementary material for: A Novel Dynamic Neonatal Blood-Brain Barrier on a Chip
Source: PLoS One. 2015 Nov 10;10(11):e0142725. doi: 10.1371/journal.pone.0142725 (PMC4640840; doi:10.1371/journal.pone.0142725)
Supplement: S1 Table — (DOCX) [file pone.0142725.s005.docx]

**S1 Table: Raw values of electrical resistance for B^3^C and transwell on the day of permeability measurement (post culture time of 5 days).**

| Experimental Condition | Electrical Resistance in B3C (Ω x 10^3^) | Electrical Resistance in transwell (Ω.cm^2^) |
| --- | --- | --- |
| Cell-free | 101.7 ± 13.7 | 123.57 ± 2.8 |
| RBEC | 177.0 ± 12.9 | 155.7 ± 3.4 |
| RBEC + ACM | 252.0 ± 20.7 | 171.7 ± 1.7 |
